# Histologic evidence of the effect of fibroblast growth factor 2 on periodontal regeneration: A scoping review of animal studies

**DOI:** 10.34172/japid.2025.3487

**Published:** 2025-02-12

**Authors:** Fazele Atarbashi-Moghadam, Saede Atarbashi-Moghadam, Termeh Sarrafan Sadeghi, Niloofar Taghipour, Ali Azadi

**Affiliations:** ^1^Department of Periodontics, Dental School, Shahid Beheshti University of Medical Sciences, Tehran, Iran; ^2^Dental Research Center, Research Institute of Dental Sciences, Shahid Beheshti University of Medical Sciences, Tehran, Iran; ^3^Department of Oral and Maxillofacial Pathology, Dental School, Shahid Beheshti University of Medical Sciences, Tehran, Iran; ^4^Department of Tissue Engineering and Applied Cell Sciences, School of Advanced Technologies in Medicine, Shahid Beheshti University of Medical Sciences, Tehran, Iran; ^5^Research Fellow, Dentofacial Deformities Research Center, Research Institute for Dental Sciences, Shahid Beheshti University of Medical Sciences, Tehran, Iran

**Keywords:** Animal models, Fibroblast growth factor 2, Periodontal diseases, Review

## Abstract

**Background.:**

Fibroblast growth factor 2 (FGF2) is a signaling molecule used successfully in periodontal regeneration. This review aims to evaluate histologic evidence of the effect of FGF2 on the regeneration of periodontal ligament (PDL), cementum, and alveolar bone in animal studies.

**Methods.:**

A scoping review of the animal models was conducted to assess the histologic evidence of the effect of FGF2 on periodontal regeneration. The search was performed for English articles published until January 1, 2025. Any histologic findings regarding PDL, cementum, or bone regeneration and other outcomes such as epithelial down-growth, ankylosis, neovascularization, root resorption, and any clinical observation through histologic or radiographic analysis were considered as desired outcomes.

**Results.:**

The MEDLINE and Scopus databases were searched, and 516 records were identified. After the screening, 22 articles met the inclusion criteria and were included in the study. The primary outcomes measured were any histologic findings regarding the regeneration of PDL, cementum, and alveolar bone. The included studies investigated the effect of FGF2 on the various periodontal defects, including 1-, 2-, and 3-wall vertical defects, circumferential defects, furcation involvement, and recession-type defects. In all types of defects, PDL, cementum, and alveolar bone formation were enhanced in most groups containing FGF2 compared to groups without FGF2. Most studies mentioned better radiographic results regarding bone formation or bone fill.

**Conclusion.:**

FGF2 can promote regeneration in all parts of periodontal tissue in surgically created periodontal defects in animal models, including cementum, PDL, and alveolar bone.

## Introduction

 Periodontitis is a chronic inflammatory disease of tooth-supporting tissues that results in attachment loss and bone loss.^1^ The bone loss results in different osseous defects, such as horizontal, vertical, and furcation defects.^2^ An ideal periodontal regeneration is deﬁned as the formation of a functional new periodontal ligament (PDL), new cementum, and new alveolar bone.^3^ In the early stage of healing after periodontal treatments, invasion of the epithelial tissue into the periodontal defect makes the regeneration of other periodontal tissues difficult.^1^ Different surgical approaches, including guided tissue regeneration (GTR), application of various bone substitutes, growth factors, enamel matrix proteins, root surface demineralization, or combinations of them, have been used to achieve optimal and predictable regeneration.^4^

 Basic fibroblast growth factor (bFGF), also named fibroblast growth factor 2 (FGF2), is a cytokine that promotes cell proliferation and differentiation and angiogenesis, contributing to wound healing.^5^ The efficacy of FGF2 in periodontal regeneration has also been reported in several studies.^6-12^ Atarbashi-Moghadam et al showed that the sequential combination of FGF2 and TGF-β is effective in enhancing teno/ligamentogenic differentiation of PDL stem cells. In an animal study, Nagayasu-Tanaka et al^14^ showed that FGF2 first promoted fibroblast cell proliferation, then enhanced angiogenesis by increasing the number of blood vessels, and finally enhanced new PDL, new bone, and new cementum formation in periodontal defects. However, the changes in clinical parameters showed the effect of this growth factor on the regeneration process.^15-17^ However, observation of the pure effects of FGF2 on PDL, bone, and cementum formation and epithelial down-growth can only be achieved through histological assessment in animal studies.^18^ Therefore, this review aims to evaluate histologic evidence of the effect of FGF2 on the regeneration of PDL, cementum, and alveolar bone in animal studies.

## Methods

###  Protocol

 This review was based on Preferred Reporting Items for Systematic Reviews and Meta-analyses (PRISMA) guidelines.^19^ The available literature was analyzed qualitatively regarding the effect of FGF2 in the regeneration of different parts of the periodontium. The PICO question of this study was “What is the histologic evidence of the effect of FGF2 on the regeneration of the different parts of periodontium (including PDL, cementum, and alveolar bone) in animal models?”

###  Eligibility criteria

####  Types of studies

 All in vivo studies that used FGF2 for periodontal regeneration in the maxillofacial area were included. Studies in which the effect of FGF2 was evaluated on the regeneration of PDL, cementum, or bone were included. The included studies were limited to English-language articles. No publication date limit was imposed. However, publications, including abstracts, reviews, letters, and book chapters, were excluded.

####  Types of participants

 Any experimental periodontal defects, including vertical or horizontal bony defects, furcation involvements, or recession-type defects in healthy animal models, such as rats, dogs, and primitives, were included. Studies such as ridge augmentation or calvarial bone defects were excluded, in which only bone regeneration, without PDL or cementum, was a concern. Tooth replantation studies were also excluded. Moreover, animal models with systemic disease were excluded.

####  Types of intervention 

 Studies using FGF2 with or without combination with other components, including any growth factor or scaffold materials, were included. Studies in which the effect of FGF2 was confounded with other factors and studies without a control group were excluded.

####  Types of outcome measures 

 Any histologic findings regarding PDL, cementum, or bone regeneration and other outcomes such as epithelial down-growth, ankylosis, neovascularization, root resorption, and any clinical observation through histologic or radiographic analysis were considered as desired outcomes.

###  Information sources and search strategy

 The MEDLINE via PubMed and Scopus database was used for the electronic search. The search was run for English articles published until January 1, 2025, using the following terms: (fibroblast growth factor 2 OR basic fibroblast growth factor OR FGF2 OR bFGF OR rhFGF2) AND (periodontal regeneration OR periodontal defect OR furcation involvement OR furcation defect).

###  Study selection and data collection process

 Study selection was performed according to the search terms. Then, two authors (FAM and TSS) screened the primary titles and abstracts independently. Afterward, three authors (FAM, SAM, and NT) independently conducted full-text screening according to inclusion and exclusion criteria. Finally, three authors (FAM, TSS, and NT) independently extracted the related data into the pre-designed tables. Any conflict at any stage of the reviewing, selection, and data extraction process was resolved by the final verdict of the corresponding author after a discussion between all authors.

###  Data items

 The outcomes of the included studies were extracted and summarized in peri-designed tables using the following data items:

###  Study characteristics 

 (1) Animal model type, (2) periodontal defect type, (3) treatment groups, (4) results of radiographic and histological analysis, (5) follow-up time.

###  Outcomes

 (1) PDL regeneration, (2) cementum regeneration, (3) bone regeneration, (4) epithelial down-growth, (5) ankylosis, (6) neovascularization, (7) root resorption, and (8) any other observation.

 The tables were revised and corrected as needed during the data extraction process.

###  Risk of bias in individual studies

 SYRCLE’s risk of bias tool for animal studies ^20^ was used to assess the risk of bias in the included animal studies. However, we should note that it was not possible to consider each factor’s weight for the overall assessment. Two authors (TSS and MRR) carried out the assessment process independently, and any disagreement was resolved through conversation.

## Results

###  Characteristics of the included studies

####  Search results

**Figure 1:**
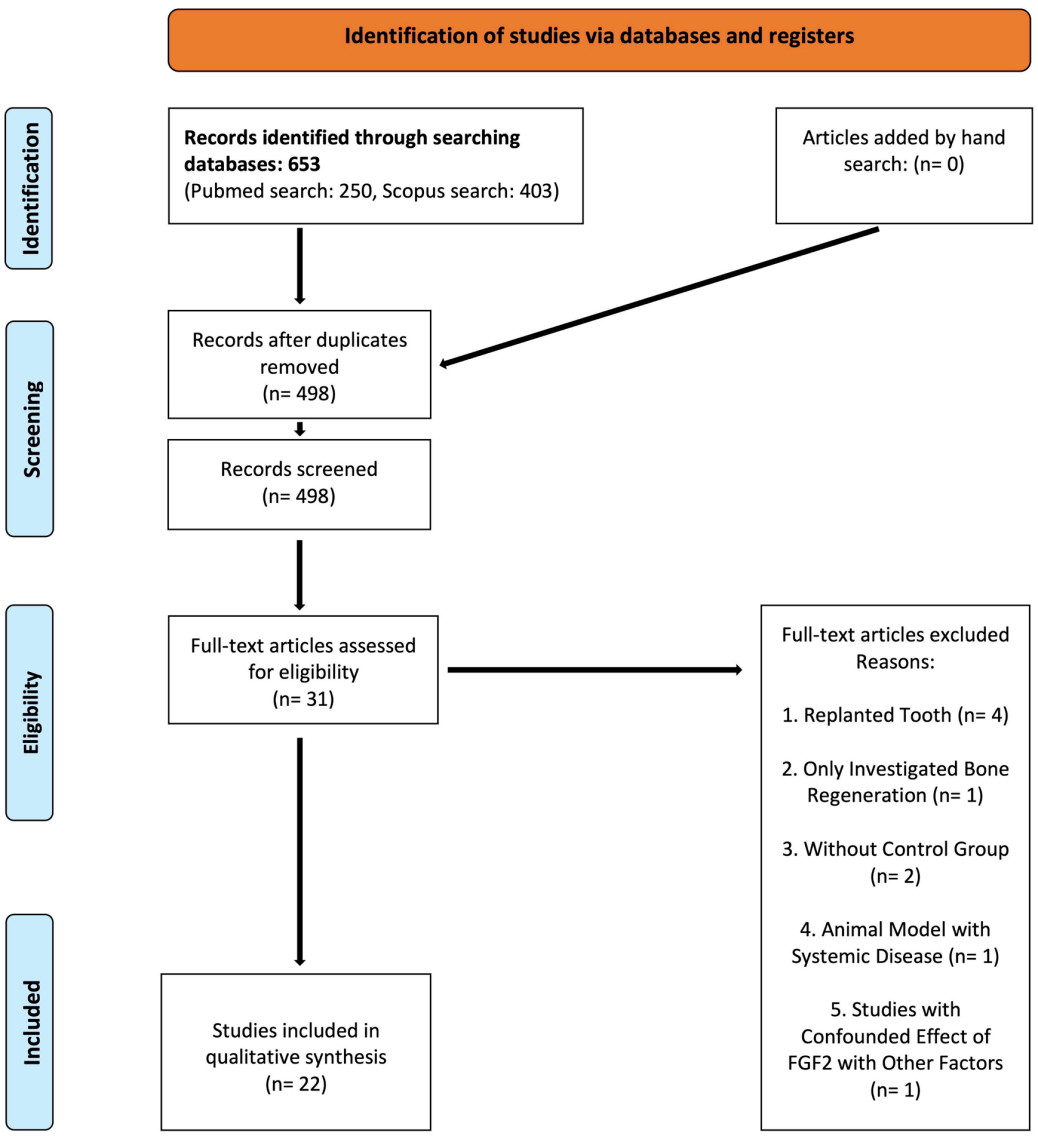
The flow diagram of the review

 After the final search, 279 articles in the MEDLINE database and 455 articles in the Scopus database were found; after eliminating duplicates, 516 articles were identified for the screening process. Twenty-two studies were included and selected for the qualitative synthesis after screening and assessing the eligibility of records (Figure 1). Tables 1, 2, and 3 summarize the extracted data of the included studies regarding the type of the investigated periodontal defects.

**Table 1:** In vivo studies that used FGF2 for periodontal regeneration in horizontal and vertical defects

**Ref**	**Animal model**		**Treatment groups **	**Outcomes**					
	**Species /teeth**	**Periodontal defects type**		**Radiographic analysis**	**Histology/Histomorphometric analysis**				
					**epithelial down-growth**	**Cementum/cementum-like formation **	**PDL/PDL-like tissue formation **	**Bone formation **	**Other outcomes**
Nagayasu-Tanaka et al, 2022^7^	Beagle dogs/ mandibular first molar	1-wall intrabony vertical defect (4 × 4 mm)	-HPC -HPC/CO_3_Ap -HPC/CO_3_Ap/FGF2 (0.3%)	**µ CT:** Significantly higher new bone volume in HPC/CO_3_Ap/FGF2 after 24 W	NM	No significant between HPC/CO_3_Ap/FGF2 and HPC/CO_3_Ap after 24 weeks.	No significant between HPC/CO_3_Ap/FGF2 and HPC/CO_3_Ap after 24 weeks.	No significant between HPC/CO_3_Ap/FGF2 and HPC/CO_3_Ap after 24 weeks.	**Remaining scaffold:** Remaining CO_3_Ap were significantly higher in HPC/CO_3_Ap after 6 and 24 W **Ankylosis:** not identified **Root resorption:** not identified
Murakami et al, 2021^6^	Wistar rats/ maxillary first molars	3-wall intrabony vertical defect (2.0 × 2.0 × 1.7 mm)	- FGF2 (0.3%) in HCP - DBBM - FGF-2 + DBBM -Negative control	**µ CT:** Significantly higher new bone volume in FGF2 and FGF-2 + DBBM groups after 4 W	Significantly less in FGF2 and FGF-2 + DBBM groups after 4 W	Only seen in FGF2 and FGF-2 + DBBM groups after 4W	Significantly higher in FGF2 and FGF-2 + DBBM groups after 4W	Higher in FGF2 and FGF-2 + DBBM groups after 4W	**Remaining scaffold: **graft particles were encapsulated by ﬁbrous connective tissue with mild inﬂammation in the DBBM group, whereas in the FGF-2 + DBBM group, were incorporated in new bone **Ankylosis:** not identified **Root resorption:** not identified
Lee et al, 2017^30^	Mongrel dogs/ mandibular first molars	1-wall intrabony defects (5 × 4 × 4 mm)	-CP/BMP2 -CP/BMP2 + topical FGF2 (20µg)	**µ CT:** No significant difference in NBF and remaining scaffold after 4 and 8W.	NM	Significantly increased in topical FGF2 application after 8W.	Not significant between groups	Significantly increased in topical FGF2 application after 8W.	**Remaining scaffold:** significantly reduced in topical FGF2 application after 8W. **Osteoclast: **significantly increased in topical FGF2 application after 8W. **Neovascularization:** significantly increased in topical FGF2 application after 8W. **Root resorption**: not identified
Matsuse et al, 2017^16^	Beagle dog/ mandibular second and fourth premolars	2-wall intrabony defects (5 × 6 × 4 mm)	- αTCP - αTCP/immobilized FGF2 (immersing in 1 mg/ml stock)	**µ CT: ** BMC was higher in the FGF2 group from 2 to 8W. This difference was significant at 2 and 4W.	NM	Significantly higher in FGF2 group until 4W.	Significantly higher in the FGF2 group until 4W.	Significantly higher in the FGF2 group from 2 to 8W. At 8W, mature bone formation (Haversian structure) was observed in the FGF2 group.	**Remaining of scaffold:** absorption started earlier and was significantly reduced in FGF2. **Neovascularization:** significantly observed in the FGF2 group from 2 to 8W. **Ankylosis:** not identified
Anzai et al, 2016^27^	Beagle dog /mandibular first molars	2-wall intrabony defects (5 × 4 × 3 mm)	-FGF2 solution in 3% HPC (0.3%) - 3% HPC -Positive control (without creating defects)	**X-ray: ** BMC was significantly higher in FGF2 groups. However, in both remained unchanged from 2 to 13M. **µ CT: **NBF and CB were significantly higher in FGF2 groups. However, no difference was observed in TB after 13M. The CB and NBF of both groups were lower than those of the positive control group.	Not observed in FGF2 groups	Significantly higher in the FGF2 group after 13M.	Significantly higher in the FGF2 group after 13M.	After 13M, NBF was significantly higher in FGF2 group. TB was similar in both groups, while they were higher than the positive control.	**Ankylosis**: not identified
Ogawa et al, 2016^23^	Beagle dog /mandibular second and fourth premolars	1-wall intrabony defects (5 × 3 mm)	- nano βTCP -COL - nano βTCP/COL / immobilized FGF2 (50 µg)	**X-ray: ** Increase radiopacity in the FGF2 group after 4W	Significantly less in FGF2 group	Significantly higher in FGF2 group after 4W	Significantly higher in FGF2 group after 4W. However, there was no significant difference between the FGF2 group and βTCP alone	Significantly higher in FGF2 group at 10D and 4W	**Clinical Observation:** Signiﬁcant gingival recession in COL group **Remaining scaffold:** no residual material, including b-TCP and collagen, after 4W **Neovascularization:** Significantly observed in the FGF2 group from 10d **Ankylosis:** not identified **Root resorption:** not identified
Saito et al, 2016^21^	Beagle dog /mandibular second and third incisors	circumferential defects (Defect height from CEJ: 4 mm)	- COL - COL/BMP2(4 μg) - COL/FGF2(4 μg) - COL/FGF2(2 μg)/ BMP2(4 μg) - COL/FGF2(2 μg) + COL/BMP2 (4 μg)	NM	Significantly higher in the Col group at 8W	Significantly higher in COL/FGF2 + COL/BMP2 group at 8W	Fiber bundles attached to new cementum and regenerated were observed in COL/FGF2 and COL/FGF2 + COL/BMP2 groups at 8W, respectively.	Significantly higher in both COL/FGF2/ BMP2 and COL/FGF2 + COL/BMP2 groups at 8W	**Clinical Observation:** Signiﬁcant gingival recession in COL group **Neovascularization:** Was observed in COL/FGF2 + COL/BMP2 group after 8W **Root resorption:** Was observed in COL/ FGF2/BMP2 group **Ankylosis: **Significantlyobserved at COL/FGF2/BMP2 and COL/BMP2
Nagayasu-Tanaka et al, 2015^14^	Beagle dog /mandibular first molar	3-wall intrabony (5 × 3 × 4 mm)	-FGF2 solution in 3% HPC (0.3%) - 3% HPC	NM	NM	Significantly higher in FGF2 group at 4 weeks	Significantly higher in FGF2 group at 4 weeks	Significantly higher in FGF2 after 1, 2, and 4 weeks	**Neovascularization:** Angiogenesis was observed in newly formed granulation tissue in the FGF2 group at day 3.
Shirakata et al, 2013 ^24^	Beagle dog /mandibular second and fourth premolars	1-wall intrabony defects (5 × 5 × 5 mm)	-βTCP -βTCP/EMD -βTCP/FGF2 in 3% HPC (0.3%) -βTCP /EMD/FGF2 in 3% HPC (0.3%)	NM	Significantly less in βTCP /EMD/FGF2 group after 10W	Significantly higher in βTCP /EMD/FGF2 and βTCP/EMD groups after 10 W. However, in the FGF2 group, thick cellular intrinsic fiber cementum more frequently.	Observed at βTCP/FGF2 and βTCP /EMD/FGF2	No significance was observed.	**Remaining scaffold:** Spare and encapsulated in the new bone or connective tissue in all groups after 10W
Oortgiesen et al, 2012 ^32^	Wistar rat/ maxillary first molars	3-wall intrabony defect (2 × 2 × 1.7 mm)	-PLGA/CP - PLGA/CP + PGA/BMP2 (10 μg) - PLGA/CP + PGA/FGF2 (25 μg)	NM	Less in FGF2 group after 12W	Only observed in the FGF2 group after 12W	Significantly higher in FGF2 group after 12W	Significantly higher in BMP2 and FGF2 groups after 12W	**Remaining scaffold:** CP occasionally was observed in all groups after 12W **Root resorption:** more frequently observed in CP group after 12W
Anzai et al, 2010 ^28^	Beagle dog /mandibular first molars	1-wall intrabony defects (5 × 5 mm)	-βTCP -βTCP / FGF2 in 3% HPC (0.3%)	**X-ray: **BMC was significantly higher in FGF2 groups at 6W.	Significantly less in FGF2 group after 6W.	Significantly higher in FGF2 group after 6W.	Significantly observed in FGF2 group after 6W.	Significantly higher in FGF2 group after 6W.	**Remaining scaffold: **significantly reduced in FGF2 application after 6W. **Ankylosis:** not identified **Inflammation:** not identified
Shirakata et al, 2010 ^11^	Beagle dog /mandibular second and fourth premolars	2-wall intrabony defects (5 × 5 × 5 mm)	- EMD - PDGF/βTCP -FGF2 solution in 3% HPC (0.3%) -Negative control	**X-ray: ** Significant radiopacity FGF2 group after 8W.	Significantly less in the EMD group after 8W	Significantly higher in all groups compared to negative control after 8W. However, in the FGF2 group, thick cellular Intrinsic fiber cementum was more frequent.	The collagen fibers appeared sparser in the FGF2 group than those observed in the EMD and PDGF/βTCP groups.	Significantly higher in FGF2 group after 8W	**Bone resorption:** Host bone resorption was observed in negative control after 8W.
Oi et al, 2009 ^22^	Beagle dog /mandibular second and fourth premolars	2-wall intrabony defects (5 × 5 × 5 mm)	-βTCP -FGF2 in HPC (200 µg) - βTCP + FGF2	NM	NM	Significantly higher in βTCP + FGF2 group at 8W	Observed in both FGF2 and βTCP + FGF2 groups at 8W.	Significantly higher in βTCP + FGF2 group at 8W	**Neovascularization:** Observed in the βTCP + FGF2 group after 2 and 4W. **Ankylosis:** Not identified **Root resorption:** Not identified
Nakahara et al, 2003 ^17^	Beagle dog /maxillary and mandibular canines	3-walled defects (3 × 4 × 4 mm)	-Col I-III -Col I-III/gelatin microsphere FGF2 (100 µg)	NM	Only observed in the Col group	Observed in FGF2 group after 4W	Observed in FGF2 group after 4W	Significantly higher in FGF2 group after 2 and 4W	**Remaining of scaffold:** Gelatin microspheres remained in some parts **Neovascularization:** Observed in FGF2 group after 2 and 4W **Ankylosis:** Not identified **Root resorption:** Only observed in Col group
Murakami et al, 19998	Beagle dog / mandibular third and first molars	3-wall defects (3 × 3 × 4 mm)	- Gelatinous carrier - Gelatinous carrier /FGF2 (50 µg)	NM	Not observed in FGF2 groups	Significantly higher in FGF2 group after 4W	Significantly higher in FGF2 group after 4W	Significantly higher in FGF2 group after 2 and 4W	**Ankylosis:** not identified **Root resorption:** not identified

αTCP = alpha tricalcium phosphate; BMC = bone mineral content, BMP2 = bone morphogenic protein 2, βTCP = beta tricalcium phosphate; DBBM = Deproteinized Bovine Bone Material; CB = cortical bone; CB-FGF2 = collagen binding-fibroblast growth factor; CEJ = cementoenamel junction; COL = collagen, CP = collagen powder; D = day; EMD = enamel matrix derivative; FGF2 = fibroblast growth factor 2, GTR = guided tissue regeneration; HPC = hydroxypropyl cellulose; M = month, µ CT = micro-computed tomography, NBF = new bone formation, NM = not mentioned, PDGF = Platelet-derived growth factor; PDL = periodontal ligament, PGA = propylene glycol alginate; PLGA = poly (DL-lactic-co-glycolic acid); CO_3_Ap = Carbonated Apatite; TB = trabecular; W = week; M = month; d = days.

**Table 2:** In vivo studies that used FGF2 for periodontal regeneration in furcation involvements

**Ref**	**Animal model**		**Treatment groups **	**Outcomes**					
	**Species /teeth**	**Periodontal defects type**		**Radiographic analysis **	**Histology/Histomorphometric analysis**				
					**epithelial down-growth**	**Cementum/cementum-like formation **	**PDL/PDL-like tissue formation **	**Bone formation **	**Other outcomes**
Momose et al, 2016 ^26^	Beagle dog /mandibular second, third, and fourth premolars	Buccal class II furcation defects (5 × 3 mm)	- COL hydrogel - COL hydrogel /FGF2 (50 μg) - Negative control	**X-ray: ** Increase radiopacity in FGF2 group at 10d and 4W	Significantly less in both groups compared with negative control after 4W.	Significantly higher in FGF2 group after 4W.	Significantly higher in FGF2 group after 4W.	Significantly higher in FGF2 group after 4W	**Clinical Observation:** evidence of gingival recession in the negative control group. **Neovascularization:** significantly observed in the FGF2 group from 10d to 4W. **Ankylosis:** not identified **Root resorption:** not identified
Saito et al, 2013 ^25^	Beagle dog /mandibular premolars	Class III furcation defects (height: 4 mm)	- FGF2 (0.3%) - βTCP/ FGF2 (0.3%) -Negative control	NM	Significantly less in βTCP/ FGF2 at 8W	Significantly higher in βTCP/ FGF2 and FGF2 groups after 8W	Was as observed in both βTCP/ FGF2 and FGF2 groups after 8W	Significantly higher in the βTCP/ FGF2 group after 8W	**Remaining of scaffold: ** Small amounts of β-TCP were remained **Osteoclast: **Was observed in FGF2 groups after 8W **Neovascularization:** Was observed in βTCP/ FGF2 group after 8W **Ankylosis:** not identified **Root resorption:** not identified
Murakami et al, 2003 ^9^	Beagle dog /mandibular first molars	**Inflamed** furcation class II defects (4 × 3 mm)	- Gelatinous carrier - Gelatinous carrier /FGF2 (0.1 % or 30 µg)	NM	Not observed in FGF2 groups	Significantly higher in FGF2 group after 6W	Observed in FGF2 group after 6W	Significantly higher in FGF2 group after 6W	**Ankylosis:** Not identified **Root resorption:** Not identified
Takayama et al, 2001 ^31^	Macaca Fascicularis / maxillary and mandibular first and second molars	**Inflamed **class II furcation defects (4 × 3 mm)	- Gelatinous carrier - Gelatinous carrier FGF2 (10 µg) - Gelatinous carrier FGF2 (40 µg) -Negative control	NM	Only observed in the gelatinous carrier and negative control after 8W	Significantly higher in the 40-µg FGF2 group after 8W	Only observed in FGF2 groups after 8W	Significantly higher in the 40-µg FGF2 group after 8W	**Ankylosis:** Not identified in FGF groups **Root resorption:** Not identified in FGF groups
Rossa et al, 2000 ^15^	Mongrel dog /second and fourth premolars	Class III furcation defects (5 × 7 mm)	- GTR - GTR + FGF2 (0.5 mg) - GTR + FGF2 (1 mg)	NM	Less in FGF2 groups after 12W	Significantly higher in the 0.5-mg FGF2 group after 12W	NM	Significantly higher in the 0.5-mg FGF2 group after 12W	**Neovascularization:** Significantly observed in FGF2 groups after 12W **Root resorption:** Negligibly observed in FGF2 groups
Murakami et al, 1999 ^8^	-Beagle dog / mandibular third and fourth premolar and first molars	Class II furcation defects (4 × 3 mm)	- Gelatinous carrier - Gelatinous carrier /FGF2 (30 µg) - Negative Control	NM	Not observed in FGF2 groups	Significantly higher in FGF2 group after 6W	NM	Significantly higher in FGF2 group after 6W	**Ankylosis:** not identified **Root resorption:** not identified
	- Macaca Fascicularis firstand second molars	Class II furcation defects (4 × 3 mm)	- Gelatinous carrier -Gelatinous carrier /FGF2 (40 µg) - Negative Control			Significantly higher in FGF2 group after 8W	NM	Significantly higher in FGF2 group after 8W	

βTCP = beta tricalcium phosphate; COL = collagen; FGF2 = fibroblast growth factor 2, GTR = guided tissue regeneration; HPC = hydroxypropyl cellulose; M = month, NM = not mentioned, PDGF = Platelet-derived growth factor; PDL = periodontal ligament, W = week.

**Table 3:** In vivo studies that used FGF2 for periodontal regeneration in recession-type defects

**Ref**	**Animal model**		**Treatment groups **	**Outcomes**					
	**Species /teeth**	**Periodontal defects type**		**Radiographic analysis **	**Histology/Histomorphometric analysis**				
					**Epithelial length **	**Cementum/cementum-like tissue formation **	**PDL/PDL-like tissue formation **	**Bone formation **	**Other outcomes**
Shujaa Addin et al, 2017 ^10^	Beagle dog /maxillary canines	Removal of keratinized gingiva + dehiscence defects (5 × 6 mm)	- Gelatin/βTCP -Gelatin/βTCP/FGF2 (0.3%)	**µ CT: ** Significantly more bone volume in FGF2 group after 8W	Significantly longer in the βTCP group after 8W	Significantly higher in FGF2 after 8W	Observed in both groups after 8W. However, PDL fibers in the FGF2 group inserted perpendicularly into the new cementum	Significantly higher in FGF2 group after 8W	**Clinical observation: **Root coverage was achieved completely in both groups **Remaining scaffold: **not identified **Ankylosis:** not identified **Root resorption:** not identified
Cha et al, 2016 ^29^	Mongrel dogs / mandibular third incisors	Removal of keratinized gingiva + dehiscence defects (5 × 6 mm)	- COL - COL/FGF2	NM	No significant difference	Significantly higher in FGF2 group after 16W	NM	No significant difference	**Clinical Observation: **Root coverage is significantly higher in the FGF2 group at 4W. However, no significant difference after 16W **Cast analysis: **Root coverage was not significantly different after 16W

βTCP = Beta tricalcium phosphate, CEJ = Cementoenamel junction, COL = collagen, FGF2 = Fibroblast growth factor 2, µ CT = micro-computed tomography, PDL = Periodontal ligament, W = weeks.

###  Risk of Bias Assessment

**Figure 2:**
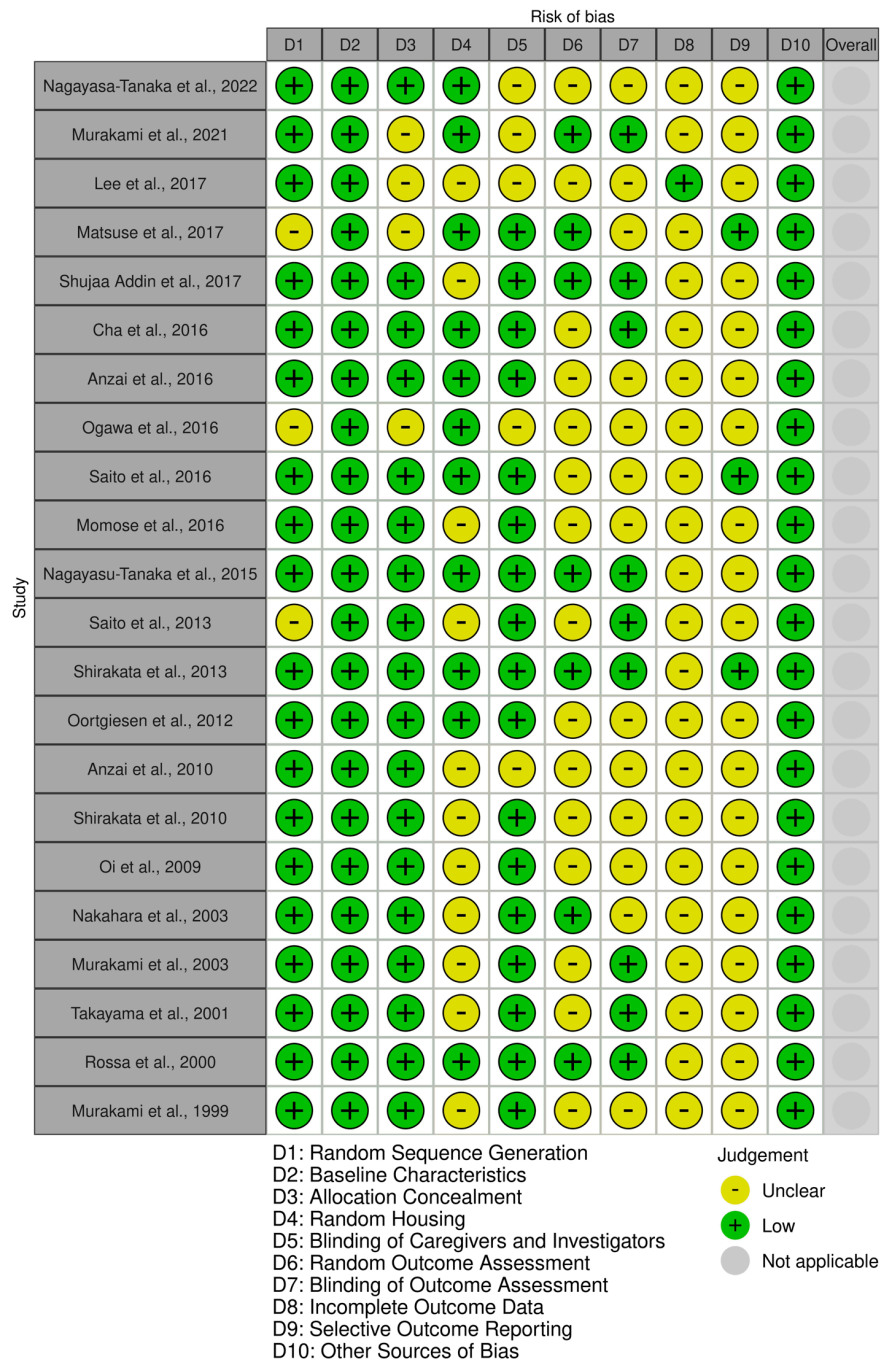
Quality assessment of the included studies according to SYRCLE

 None of the studies has a domain with a high risk of bias. All the studies exhibit a low risk of bias in the “Baseline Characteristics” domain. Moreover, most of the studies have a low risk of bias in the “Random Sequence Generation” and “Allocation Concealment” domains. Most of the studies in the “Random Outcome Assessment,” “Blinding of Outcome Assessment,” “Incomplete Outcome Data,” and “Selective Outcome Reporting” domains have an unclear risk of bias (Figure 2).

###  Characteristics of animal models

 Most of the studies used Beagle^7-11,14,16,17,21–28^ and Mongrel^15,29,30^ dogs for animal models. Only two studies used Macaca Fascicularis as models^8,31^ and Wistar rats.^6,32^

###  Intervention characteristics

####  Periodontal defects

 The effect of FGF2 was investigated on circumferential and vertical defects, furcation involvements, and recession-type defects in 15,^6–8,11,14,16,17,21-24,27,28,30,32^ six,^8,9,15,25,26,31^ and two^10,29^ studies, respectively. Murakami et al^8^ investigated the effect of FGF2 on both vertical defects and furcation involvements.

 Among the circumferential and vertical defects, one study was on circumferential defects,^21^ with five studies on 3-wall vertical defects,^6,8,14,17,32^ four studies on 2-wall vertical defects,^11,16,22,27^ and five studies on one-wall vertical defects.^7,23,24,28,30^ The dimensions of the created circumferential and vertical defects were mostly 3–6 mm. Among the furcation defects, there were four studies on class II furcation defects,^8,9,26,31^ with two studies on class III furcation defects.^15,25^ The dimensions of the created furcation defects were 3–7 mm. All studies on recession-type defects were on 5 × 6-mm dehiscence defects.^10,29^

####  Follow-up time

 The range of follow-up times in studies investigating vertical and circumferential defects was between 4 weeks and 13 months. This range was 4‒12 weeks in studies investigating furcation defects and 8‒16 weeks in studies investigating recession-type defects.

####  Carriers and combinations

 The materials used as FGF2 carriers or scaffolds were as follows: hydroxypropyl cellulose (HPC), ^6,7,11,14,22,24,27,28^ collagen materials,^17,21,23,26,29,30^ the combination of poly (DL-lactic-co-glycolic acid) (PLGA) with collagen powder and polyglycolic acid (PGA), ^32^ gelatinous carriers, ^8–10,31^ beta-tricalcium phosphate (βTCP),^10,11,22–25,28^ alpha-tricalcium phosphate (αTCP),^16^ carbonated apatite (CO_3_Ap),^7^ and deproteinized bovine bone material (DBBM) ^6^. One study combined the GTR process using FGF2.^15^

 Some included studies assessed combinations of enamel matrix derivatives (EMD)^24^ and morphogenic protein 2 (BMP2)^21,30^ with FGF2.

###  Outcomes

####  Histologic/histomorphometric analysis

####  Cementum or cementum-like tissue regeneration

 Nineteen studies mentioned that FGF2 use resulted in higher cementum or cementum-like tissue regeneration compared to groups without FGF2. ^6,8–10,14–17,21–23,25–32^

 One study showed that this parameter was significantly higher in the βTCP/ FGF2 group compared to FGF2 alone, ^22^ and one study suggested that using a combination of collagen materials with BMP2 and FGF2 simultaneously resulted in more cementogenesis than using FGF2 and BMP2 with collagen materials separately. ^21^

####  PDL or PDL-like tissue regeneration

 Fifteen studies reported higher PDL or PDL-like tissue formation in groups containing FGF2 compared to control groups.^6,8,9,14,16,17,21,22,24-28,31,32^ One study mentioned that FGF2 in combination with βTCP resulted in higher PDL or PDL-like tissue formation than collagen materials alone; however, there was no significant difference regarding PDL regeneration between the combination of βTCP with FGF2 and βTCP alone.^23^

####  Bone formation

 Eighteen studies suggested that groups with FGF2 showed significantly more new bone formation than groups not containing FGF2.^6,8-11,14-17,21-23,25-28,30,31^ Two studies suggested that the combination of βTCP with FGF2 could induce more bone formation than the βTCP and FGF2 alone,^22,25^ and one study found significantly higher bone formation in groups that contained both BMP2 and FGF2 than FGF2 or BMP2 alone.^21^

####  Epithelial down-growth

 Among vertical, circumferential, and furcation defects, 13 studies mentioned that the epithelial down-growth was lower in groups containing FGF2 compared to groups without FGF2^6,8,9,15–17,23–25,27,28,31,32^; one study mentioned that the combination of EMD with FGF2 was more effective than using them separately,^24^ one study claimed that using EMD alone was more effective than using FGF2 in decreasing the epithelial down-growth.^11^ Another study showed that this parameter was significantly lower in the βTCP/FGF2 group compared to FGF2 alone.^25^

 Regarding the epithelial length, the results were not convergent in studies on recession-type defects; one study showed βTCP results in a longer epithelium than its combination with FGF2,^10^ one study showed no significant difference between collagen materials alone or in combination with FGF2.^29^

####  Radiographic analysis

 Four studies used intraoral X-rays,^11,23,26,28^ and five studies used micro-computed tomographic (µCT) radiographs^6,7,10,16,30^ for radiographic examination; one study used both µCT and intraoral X-rays.^27^ The other thirteen studies did not mention anything about radiographic analysis. Among studies with radiographic analysis, nine studies reported higher bone mineral contents or new bone formation in experimental groups containing FGF2 compared to groups without FGF2. ^6,7,10,11,16,23,26–28^ Nevertheless, one study showed no significant difference between using a combination of collagen powder and BMP2, with or without FGF2, in new bone formation.^30^

## Discussion

###  Summary of findings

**Figure 3:**
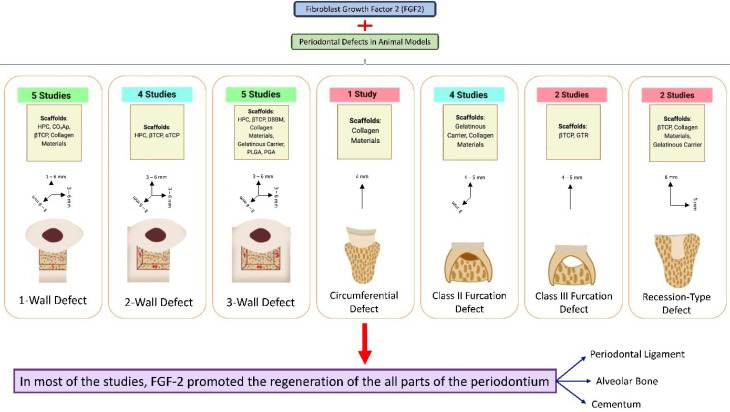
Summary of the study’s findings. There were seven different types of periodontal defects explored in the included studies: one-wall defect,^7,23,24,28,30^ 2-wall defect,^11,16,22,27^ 3-wall defect, ^6,8,14,17,32^ circumferential defect,^21^ class II furcation defect,^8,9,26,31^ class III furcation defect, ^15,25^ and recession-type defect.^10,29^ For each type of defect, a column represents the number of studies conducted on that kind of defect, the scaffolds used to carry fibroblast growth factor 2 to that defect, and the reported range of dimensions for that defect in the studies

 The present study assessed the histological findings regarding the FGF2 potential in the true regeneration of periodontal tissues (cementum, PDL, and bone). Figure 3 depicts the summary of the findings of the present review. Except for the amount of reduction in the epithelial down-growth of the recession-type defects (which was inconclusive), overall, FGF2 increased the amount of regenerated PDL, bone, and cementum and reduced epithelial down-growth (other defect types) in the periodontal defects compared to the equivalent treatment without FGF2. The most used animal model in all studies was the beagle dog. Considering all used carriers and scaffolds for transferring FGF2 to the defect area, βTCP and DBBM elicited the most capability for enhancing the actual regeneration of periodontium.

###  Interpretation of the results

 Healing of intrabony periodontal defects is associated with the number of bone walls, which determine the extent and location of available cells and vascularity and factors influencing regenerative potential.^33^ One-wall vertical periodontal defects have a more problematic and complex regeneration process than two- or three-wall defects^33^; hence, using one-wall defect models can extensively reveal the effect of the intervention.^30^ This review showed that applying FGF2 had a promising potential for regenerating periodontal tissue even in one-wall defects.^7,23,24,28,29^ These findings could help further clinical investigations and suggest a successful treatment plan for one-wall vertical periodontal defects. A grade III furcation involvement is the most difficult periodontal defect in achieving periodontal regeneration.^25^ This study found that in furcation defects (grade II and III), FGF2 could enhance bone and cementum formation and PDL regeneration and inhibit epithelium down-growth.^9,15,25,26^ Probably, the potential of FGF2 to increase cell proliferation and angiogenesis promotes the healing process even in non-contained defects and enhances periodontal regeneration.^23-25,28^

 The regeneration process is complicated in recession-type periodontal defects because of the difficulty of space maintenance.^10^ However, studies on this issue are limited, and only two studies met the inclusion criteria of this review.^10,29^ Both studies showed significantly higher cementum formation in FGF2 groups, but the evidence about bone formation was controversial. Cha et al^29^ showed significantly more root coverage in the FGF2 group at four weeks, but after 16 weeks, there was no significant difference, suggesting that FGF2 accelerates the healing process. Some in vivo studies examined the coronally advanced flaps alone or in combination with connective-tissue grafts (CTGs) to heal the recession-type defects; nevertheless, no periodontal regeneration was observed after nine months in these studies, with just long junctional epithelium attachment alongside limited amounts of cementum and bone regeneration.^34-38^ Therefore, applying FGF2, which results in the regeneration of all parts of periodontal tissue, can be beneficial for a possible reconstruction of recession defects.

 The EMD^24^ and BMP2^21,30^ were combined with FGF2 in some studies. EMD showed a higher preventive effect on the epithelium down-growth, whether combined with FGF2 or used alone.^11^ BMP2 has shown higher potential for bone regeneration when combined with appropriate amounts of FGF2 than when used alone. Moreover, FGF2 and BMP2 with a bilayer design were used to regenerate periodontal defects, with promising results in the regeneration of alveolar bone and PDL and cementum.^21^

 As FGF2 is a solution, most studies used carriers or scaffolds to preserve this growth factor in the treated areas.^6,7,16,22,23^ Using βTCP as a carrier for FGF2 showed a higher potential for stimulating the regeneration of periodontal tissues^22,25,39^ possibly because of the tunnel structure of the βTCP, making it a scaffold for bone formation and vascularization. Furthermore, the βTCP can entrap the FGF2 inside it and prevent the early diffusion of FGF2. Thus, it would make higher concentrations of FGF2 available for periodontal tissue regeneration. The particle size of the scaffold used can also affect the amounts of remaining materials after the healing period and bone formation. It may be possible to claim that particles with greater size can stay longer in the defect area and affect the regeneration process with their osteoinductive and osteoconductive features. Hence, DBBM showed a higher potential for periodontal tissue regeneration than other scaffolds in the Shirakata et al study.^40^

 Animal studies are essential before the clinical phase to evaluate various medications and treatment modalities. Moreover, selecting the best animal model according to research objectives is vital.^18^ Numerous animals from different species have been used in studies related to periodontal disease and regenerative treatments.^41^ However, it is difficult to determine whether the outcomes can be applied to human clinics due to the difference between the pathogenesis of periodontal disease and the response to treatment modality, although large animals exhibit more human-like characteristics.^18^ As a model for assessing periodontal disease, dogs are extremely valuable models in terms of periodontal tissues and tooth size comparable to humans.^18,41^ In addition, periodontal microbiota in their supragingival and subgingival regions are similar to those in humans.^18^ Also, due to its size and cooperative nature, the beagle dog is one of the most commonly used breeds.^41^ According to our review, 16 studies have been conducted on beagle dogs, with three studies on mongrel dogs.

## Limitations and recommendations for future studies

 This study has its limitations. First, there was no study with a low risk of bias in all domains; the domains with an unclear risk of bias can negatively influence the certainty of the evidence. None of the studies compared the effect of FGF2 on different intrabony defects, like the comparison between grade II and III of the furcation involvements or one-wall with two-wall vertical defects. Therefore, a true comparison between the effects of FGF2 on different periodontal defects cannot be accomplished. Furthermore, a reliable meta-analysis could not be achieved due to the variety of reporting methods, type of defects, and follow-up time of studies. Eventually, this review has been conducted on animal studies to evaluate the effect of FGF2 on different components of periodontium histologically; hence, the results cannot be completely applied to humans, and there is still a need for controlled trials on human subjects in this field.

## Conclusion

 In conclusion, FGF2 can promote regeneration in all parts of periodontal tissue in surgically created periodontal defects in animal models, including cementum, PDL, and alveolar bone. Moreover, βTCP and DBBM demonstrated the most potential for enhancing periodontal regeneration among all the scaffolds and carriers used to transfer FGF2.

## Competing Interests

 The authors declare no known competing financial interests or personal relationships that could have appeared to influence the work reported in this paper.

## Data Availability Statement

 Data supporting this study are included within the article and/or supporting materials.

## Ethical Approval

 Not applicable.
